# Changes in Urinary Phthalate Metabolite Levels Before and After the Phthalate Contamination Event and Identification of Exposure Sources in a Cohort of Taiwanese Children

**DOI:** 10.3390/ijerph14080935

**Published:** 2017-08-19

**Authors:** Chian-Feng Huang, I-Jen Wang

**Affiliations:** 1 Institute of Epidemiology and Preventive Medicine, College of Public Health, National Taiwan University, Taipei 10055, Taiwan; davidbee416@gmail.com; 2 Taoyuan Psychiatric Center, Ministry of Health and Welfare, Taoyuan 33058, Taiwan; 3 Department of Pediatrics, Taipei Hospital, Ministry of Health and Welfare, Taipei 11267, Taiwan; 4 Institute of Environmental & Occupational Health Sciences, School of Medicine, National Yang-Ming University, Taipei 100044, Taiwan; 5 Department of Health Risk Management, China Medical University, Taichung 110001, Taiwan

**Keywords:** phthalate metabolite, DEHP, food contamination event, plastic bag

## Abstract

In 2011, the Taiwan Food and Drug Administration inadvertently discovered that, for decades, manufacturers had replaced expensive natural emulsifiers in food products with diethylhexyl phthalate (DEHP). We wanted to compare urinary phthalate metabolite levels of children before and after the DEHP food contamination event and identify source(s) of phthalate exposure in addition to the illegal food additives. In the present study, morning urine samples were collected from a cohort of 453 children in 2010 in Taipei. After the DEHP food contamination event, there were 200 cohort children left at follow-up in 2013. The geometric means (GMs) of urinary mono(2-ethyl-5-hydroxyhexyl) phthalate (5OH-MEHP) levels before and after the event were 9.39 and 13.34 µg/g of creatinine, respectively, with no significant difference (*p* = 0.093). After the DEHP food contamination event, we found that urinary phthalate metabolite levels were significantly higher in people who frequently consumed microwave-heated food and used fragrance-containing products (*p* < 0.05). In addition, children who did not frequently wash hands before eating had significantly higher urinary phthalate metabolite levels than those who did (*p* < 0.05). These results demonstrate that urinary phthalate metabolite levels did not decrease after the DEHP food contamination event, thus, other sources must contribute to phthalate exposure in daily life. Public awareness of approaches to reducing phthalate exposure is necessary.

## 1. Introduction

In 2011, the Taiwan Food and Drug Administration (TFDA) inadvertently discovered phthalates in food in a food contamination incident. For decades, manufacturers had replaced expensive natural emulsifiers (e.g., palm oil) with diethylhexyl phthalate (DEHP), one of the most commonly used phthalates, in many food products [1]. For the general population, the amount of DEHP would easily exceed the safety guideline per day even after consuming just one bottle of contaminated sports drink [2]. In addition to the elimination of DEHP-containing additives and contaminated products, the DEHP food contamination event raised public awareness about phthalate exposure. Because of the large-scale use of plastic products in many industries, several goods are a potential source of residual phthalates, such as toys, pharmaceuticals, and plastic bags [3].

Phthalate esters (phthalates), widely referred to as plasticizers, are industrial chemicals added to plastics for enhancing flexibility and resilience. Dietary sources are the major exposure route for the general population, for example, through leaching from food packaging material. In addition, people are continually exposed to phthalates through dust inhalation and skin contact [4]. In addition to DEHP, high-molecular weight phthalates, such as butylbenzyl phthalate (BBzP), are primarily used for polyvinyl chloride (PVC) building materials and floorings. Low-molecular weight phthalates, such as dibutyl phthalate (DBP) and diethyl phthalate (DEP), are also widely used and can be found in shampoos, lotions, and other personal care products [5].

Many studies have shown that phthalate exposure results in various adverse health outcomes. Adverse developmental, hepatocarcinogenic, endocrine, and reproductive effects were reported [6]. These endocrine-disrupting chemicals may increase the risk of breast cancer and other hormone-related cancers in adults [7]. Children, whose tolerable daily intake (TDI) is relatively lower than the TDI of adults, may be affected more adversely. Exposure of DEHP and DBP might be associated with higher risk of precocious puberty in girls [8]. In addition, girls may have an elevated risk of breast cancer and cardiovascular disease in the future [9,10]. Boys with a long-term phthalate exposure are prone to feminization. The reproductive toxicity of phthalates would shorten the penile size and anogenital distance [11] and even reduce sperm activity [12], which might result in infertility [13]. Bornehag and Nanberg [14] revealed the adjuvant effects of phthalate on Th2 differentiation, which causes a tendency toward atopic diseases. Children with excessive exposure to DEHP easily acquired atopic dermatitis and other atopic disorders [15]. Furthermore, mental disorders were found associated with phthalate exposure as well [16,17].

After the 2011 DEHP food contamination event, professional health providers urgently sought to identify the sources of phthalate exposure and follow up children to determine harm. We assumed similar responsibility and conducted a population-based cohort study on children who were at risk of phthalate exposure from 2011 to 2013. Although phthalate-contaminated food items were removed from the market during the event, many risk factors for phthalate exposure remained. For future preventive interventions, we aimed to detect other high risk factors in the daily life of children. The lessons learned in Taiwan can help many countries in long-term policymaking.

## 2. Materials and Methods

### 2.1. Participants and Information Collection

Urine samples of 453 children, aged 3 years, were collected in 2010 in Taipei; these children were selected from the Childhood Environment and Allergic Diseases Study (CEAS) cohort (Table S1). Enrollment in the study required the completion of phthalate exposure monitoring of urinary phthalate metabolite levels. Exclusion criteria included congenital disorders, chronic diseases, or inability to answer questions in Mandarin Chinese. Parents were interviewed in clinics by using a standardized questionnaire regarding child birth history, parental age and education levels, family income, parental history of atopic diseases, breastfeeding duration, and source of environmental phthalate exposure in children. In 2013 (after the 2011 DEHP food contamination event), the children were followed up at the clinics of Taipei Hospital, Ministry of Health and Welfare for the subsequent development of asthma. Two hundred children completed the follow-up, and their blood and urine specimens were available. Written informed consent was obtained from all the parents. The Institutional Review Board of the hospital approved the study protocol (TH-IRB-11-02), which complied with the principles of the Helsinki Declaration.

### 2.2. Questionnaire on Environmental Exposures

Through a 5-category scale (everyday, usually, more than once per week, rarely, and never), the parents of the participant children indicated the frequency of feeding their children hot food packed in plastic bags, food stored or heated in PVC films, microwave-heated food, drinks from plastic bottles, and instant noodles. In addition, answers regarding the frequency of hand washing before meals were collected. Parents were also asked about the frequency of using plastic nursing bottles or nipples and fragrance-containing products for children. Questions related to other environmental exposures, such as PVC flooring, incense burning at home, environmental tobacco smoke (ETS), and damp conditions, were asked in the questionnaire. In addition, we requested the parents to answer whether their children were exposed to the five contaminated food categories announced by the TFDA [18]. If the children were exposed to any contaminated food category, the parents would choose one primary contaminated food item from the following: juice and jelly, sports drink, and probiotic and vitamin supplements.

### 2.3. Urine Sample Preparation and Measurement

First mid-stream urine in the morning were collected into glass containers and stored at −20 °C until analysis. Four phthalate metabolites [monoethyl phthalate (MEP), monobutyl phthalate (MBP), monobenzyl phthalate (MBzP), and mono (2-ethyl-5-hydroxyhexyl)phthalate (5OH-MEHP)] representing exposure to four commonly used phthalates [diethylphthalate (DEP), dibutyl phthalate (DBP), butylbenzyl phthalate (BBzP), and di(2-ethylhexyl) phthalate (DEHP)], respectively, were measured using ultra-performance liquid chromatography coupled with tandem mass spectrometry (UPLC-MS/MS) (Waters, Milford, MA, USA) as described previously [19]. The limits of detection for MEP, MBP, MBzP, 5OH-MEHP were 3.27, 0.95, 0.15, and 1.36 ng/mL, respectively. For concentrations below the detection limits, a value of half the lower limit of detection was assigned. All concentrations are based on duplicate analysis. Regarding the procedure for avoiding contamination, the adsorbent was washed with twice of 0.1% NH_4_OH in methanol and twice of Milli-Q water. All glassware was rinsed with acetone and methanol sequentially after washing and before the use. All adsorbent plate was disposed after the extraction. Each batch of samples contained a reagent blank, matrix blank, two matrix spike samples, sample duplicate, and sample spike. No contamination of analytes was identified. Urinary creatinine levels were analyzed using an enzymatic assay according to manufacturer instructions [20]. All phthalate metabolite concentrations were adjusted for urinary creatinine levels.

### 2.4. Statistical Analysis

The estimated geometric mean (GM) and the geometric standard deviation (GSD) of metabolites were obtained. Because of the skewed distributions, the data were log (Ln)-transformed before further statistical tests. All log-transformed data in the study showed a normal distribution and no significant outliers were found. The urinary phthalate metabolite levels before and after the DEHP food contamination event were compared using paired t tests. In addition, the association between urinary phthalate metabolite levels and the source of environmental exposure was evaluated using t tests. Results with p < 0.05 were considered significant. All statistical analyses were performed using SPSS version 21 (SAS Institute Inc., Cary, NC, USA).

## 3. Results

### 3.1. Basic Demographics of the Study Population

Table 1 shows the baseline characteristics of the 453 participants at the first collection of urine samples in 2010. Except for maternal nationality, maternal history revealed no significant differences in the levels of the four urinary phthalate metabolites: MEP, MBP, MBzP, and 5OH-MEHP, among maternal age, education level, and atopic disease history. Additionally, Table 1 shows that the children of women with a parity of lower than 2 had higher MBP and MBzP levels. Regarding breast feeding, sex, birth weight, gestational age, and family income, no significant differences were observed among the four phthalate metabolite levels.

### 3.2. Comparison of Metabolite Levels Before and After the DEHP Food Contamination Event

**Figure 1:**
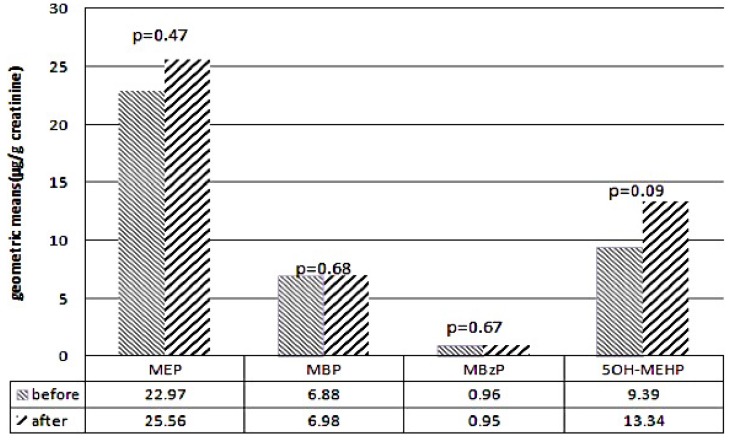
Levels of urinary phthalate metabolites before and after DEHP contaminated food event.

The GMs of urinary 5OH-MEHP levels before and after the DEHP food contamination event were 9.39 and 13.34 µg/g of creatinine (*p* = 0.093), respectively. The GMs and GSDs were applied to test the difference in the urine specimens before and after the event. Paired t tests showed no significant differences before and after the event in the levels of the four urinary phthalate metabolites: MEP, MBP, MBzP, and 5OH-MEHP, respectively (Figure 1). Before the event, the GM of the urinary MEP level (22.97 µg/g of creatinine) was higher than those of the urinary 5OH-MEHP (9.39 µg/g of creatinine), MBP (6.88 µg/g of creatinine), and MBzP (0.96 µg/g of creatinine) levels.

### 3.3. Environmental Risk Factors for Higher Urinary Phthalate Metabolite Levels

Table 2 shows several candidate environmental factors for higher urinary phthalate metabolite levels. More than 50% of the 453 children were exposed to the following factors: hot food packaged in plastic bags (79.5%), food stored or heated in PVC film (57.6%), microwave-heated food (58.4%), drinks from plastic bottles (90.9%), instant noodles (66.6%), incense burning at home (52.7%), and ETS exposure (53.2%). Children who usually consumed microwave-heated food or drinks from plastic bottles had significantly higher urinary MBP (*p* = 0.02) or MEP (*p* < 0.01) levels, respectively, than those who did not. Use of fragrance-containing products significantly increased urinary MEP levels (*p* = 0.02). We discovered that hand washing was a protective factor. Children who seldom washed hands before eating (3.7%) had significantly higher urinary MBzP levels than did those who washed their hands frequently (*p* = 0.04).

During the food scandal in 2011, the Taiwan FDA discovered that manufacturers had used DEHP to replace palm oil as a clouding agent in many types of food, which were checked for exposure. Table 3 shows the percentage of children who were exposed to such high-risk foods. According to ANOVA analysis, the level of 5OH-MEHP, a major DEHP metabolite , was a significant variable (*p* < 0.001). According to the post hoc analysis, only probiotics and vitamin supplements were significant sources of DEHP contamination (*p* < 0.0001).

We observed no significant changes in the exposure percentage of each environmental risk factor acquired after the follow-up in 2013 (Table 4). Microwave-heated food and fragrance-containing products remained significant risk factors for relatively high urinary phthalate metabolite levels, whereas hand washing was a protective factor. However, no more significant differences were observed after the event in urinary phthalate metabolite levels between children who usually consumed drinks from plastic bottles and those who did not. Nevertheless, PVC flooring became a significant environmental factor after the event that led to significantly higher urinary MBP (*p* = 0.03) and MBzP (*p* = 0.04) levels.

## 4. Discussion

The present study revealed no significant differences in urinary phthalate metabolite levels in community children after the DEHP food contamination event. This primary result suggests that some risk factors were not successfully eliminated. We found that microwave-heated foods, fragrance-containing products, and rarely washing hands are major risk factors associated with high phthalate exposure. These findings will not only help initiate additional intervention studies but also guide potential public health policies for the population.

We found no significant differences in urinary DEHP levels in children from the general population before and after the event. By contrast, Wu et al. [21] derived an opposite finding. They followed up 29 children for 6 months after the DEHP food contamination event and revealed that three urinary oxidative DEHP metabolites, 5OH-MEHP, 5oxo-MEHP, and 5cx-MEPP, positively correlated with the daily DEHP intake, and had significantly decreased compared with the baseline. However, the participants were a high-risk population from screening clinics for the event. Moreover, Wu et al. conducted an intervention for educating the involved families to avoid environmental toxicants.

Previous studies have showed that human phthalate exposure is mostly through air, food and direct skin contact. Subedi et al. [22] described the factors of high concentration of DEHP exposure through indoor dust, such as the volume, the exchange rate, temperature, moisture and interior material. In addition to contaminated foods, oils and cream-based diet were found related to high DEHP concentration by Serrano and her colleagues [23]. Other routes, such as mouthing toys and other phthalate-contained products, are also important for children to high phthalate exposure [24].

For the general population, food with packaging that contains phthalates may play one of the most essential roles. Leaching from plastic containers is the main source of DEHP exposure according to previous studies [25]. In the present research, we found that microwave-heated food had a significant association with high urinary phthalate metabolite levels before and after the event. This result implies that awareness of food containers used in heating food in a microwave is vital [26]. Plastic bags and PVC films are widely used for covering food; consequently, plasticizers in such bags and films would be easily released in food in the high temperature environment of a microwave [27,28]. Therefore, people should avoid using plastic containers for heating foods and drinks. Our study result also supports the regulation of plastic food containers and PVC films used for covering food.

Fragrance-containing products were another persistent significant risk factor found in this study. People who used such products had higher urinary phthalate metabolite levels, particularly those of MEP, a DEP metabolite, than those who did not. Similarly, Guo et al. [29] showed that using personal care products (PCPs) leads to high DEP exposure. In contrast to DEHP, DEP and DBP are low-molecular weight phthalates that persist in the environment even after contamination. Therefore, caution is necessary in using body lotions, shampoos, and other PCPs, particularly by vulnerable groups [30]. Lower thyroid hormone levels were found in pregnant women with high MBP exposure [31], which may affect the brain development of children and result in relatively low IQs [32]. In addition, increased phthalate metabolite levels showed a positive association with the risk of male congenital anomalies of genital organs [33]. Thus, for safety reasons, we advise pregnant women against using nail polishes, perfumes, and other fragrance-containing products.

We demonstrated that “drinks from plastic bottles” were no longer a significant risk factor in 2013 (Table 4). The reason for this finding may be that most types of DEHP-contaminated beverage were packaged in plastic bottles, which were removed from the market by order of the Taiwan government [18] during the DEHP food contamination event. Consequently, environmental factors played major roles. For instance, PVC flooring became a significant factor associated with higher MBP and MBzP levels in the follow-up (Table 2 and Table 4). Carlstedt et al. [34] determined a similar finding that infants in bedrooms with PVC flooring have significantly high urinary MBzP levels. Second, both “microwave-heated food” and “fragrance-containing products” became significant factors for high urinary 5OH-MEHP levels after the DEHP food contamination event.

Notably, our research shows that washing hands before eating was a persistent protective factor (Table 2 and Table 4). Children can easily reach some commercial phthalate-containing goods, such as toys and PVC flooring [3]. Thus, children are easily exposed to phthalate contamination if they eat without washing their hands after handling such phthalate-containing goods. Parents must keep children away from PVC products and remind them to wash hands frequently. Nevertheless, phthalate plasticizers can evaporate into the atmosphere without difficulty [35]. Children acquire phthalates not only from food but also through inhalation and dermal absorption, which showed an association with atopic disorders [36]. A vacuum cleaner may help remove indoor dust, which contains DEHP, BBP, and DBP [36].

During the 2011 event, phthalates were detected as tainted ingredients in many types of food [1]. In this study, we demonstrated that urinary levels of 5OH-MEHP, a major DEHP metabolite, were significantly higher after the event. Our results demonstrate that probiotics and vitamin supplements were the most DEHP-contaminated source with spiking levels of 5OH-MEHP. Consistent with our results, two popular probiotics had the highest DEHP levels among all DEHP-contaminated foodstuffs [1] during the event, according to official examinations. Although phthalates such as DBP and DEP are used as excipients to stabilize drug products [37], TFDA regulations do not allow DEHP to be added in probiotics and vitamin supplements [18]. Manufacturers illegally use DEHP to replace natural palm oil as a clouding agent for enhancing turbidity and appearance. Because of manufacturing procedures, flavor powders added in dietary supplements may be another major factor for DEHP exposure. Thus, we do not recommend excessive intake of dietary supplements by children.

In our participants, urinary MEP levels were higher than urinary MBP, MBzP, and 5OH-MEHP levels (Table 1). By comparison, some studies in Taiwan [5,38] reported higher urinary MBP levels. In addition, urinary 5OH-MEHP levels were relatively higher in Taiwanese children than in those in the United States and Germany [4,39]. This finding reflects the DEHP food-contamination event revealed in 2011. Although absolute values may vary because of different laboratory methods and systemic errors, the composition of phthalate metabolites can represent the exposure condition for the population. The inconsistencies in results because of different nationalities may be explained by different life styles, dietary habits, and environmental factors.

Our study has some potential limitations. First, some of our participants were lost to follow-up, but the remaining patients were still representative. Second, we used only 5OH-MEHP as a substitute for DEHP detection, although there are numerous DEHP metabolites in urine. However, four main secondary metabolites have been viewed by previous reports as good parameters to monitor DEHP: mono-(2-ethyl-5-hexyl) phthalate (MEHP), mono-(2-ethyl-5-oxohexyl) phthalate (MEOHP), mono-(2-ethyl-5-hydroxyhexyl) phthalate (MEHHP) and mono-(2-ethyl-5-carboxypentyl) phthalate (MECPP) [4]. In addition, 5OH-MEHP is relatively high sensitivity and remains in a proportion of the four important DEHP metabolites [40]. Thus, the choice of 5OH-MEHP as an indicator was reasonable and cost-effective. Thirdly, we were unable to control all the factors leading to high urine phthalate levels mentioned in past researches. It may impact the interpretability of our finding because the categorization might be linked to another confounding factor associated with unknown phthalate exposure. However, some of listed environmental factors in the present study were repeatedly found significant in the same cohort in 2010 and 2013, which made the findings more reliable. Furthermore, the real-life situation was considerably deviated from the null when the harm effect was calculated.

Our population-based cohort included a considerable number of participants, who were followed up twice. In addition, we administered comprehensive questionnaires to identify many essential sources of phthalate exposure. These sources were common risk factors and were comparable with those in different countries and thus useful for researchers worldwide. Measuring urinary phthalate metabolites through an objective quantitative biomarker can also reduce the recall bias. In this study, the metabolites were analyzed using UPLC-MS/MS with favorable validity and reliability. The present study facilitates understanding the existing risk factors of environmental exposure. Notable, we discovered that washing hands before eating is a simple yet effective habit that protects people from chemical toxicity. Future intervention studies are necessary to determine the efficiency of hand washing. A successful example [21] showed that phthalate exposure and subsequent urinary phthalate metabolite levels can be eventually reduced through intensive monitoring and health education. Thus, relevant instructions to patients in hospitals, health education in schools, and information released from mass media are all crucial for long-term policy making.

## 5. Conclusions

We found no significant differences in urinary phthalate metabolite levels in a population-based cohort in Taiwan before and after the 2011 DEHP food contamination event. The risk factors for phthalate exposure, such as microwave-heated foods, fragrance-containing products, and the habit of rarely washing hands before eating, still remain in daily life and necessitate future intervention studies. It is important to keep raise the public awareness of the potential phthalate sources. People can reduce the harmful exposures once they understand more facts and take more protections.

## Figures and Tables

**Table 1:** Basic demographics of the study population in terms of geometric means (s.e.) of urine phthalate metabolites concentrations (*n* = 453).

Characteristics		%	GM (s.e.) MEP (µg/g Creatinine)	GM (s.e.) MBP (µg/g Creatinine)	GM (s.e.) MBzP (µg/g Creatinine)	GM (s.e.) 5OH-MEHP (µg/g Creatinine)
**Mother**						
Maternal age	<34 years	82.2	21.98 (3.71)	5.99 (3.32)	0.95 (2.92)	9.03 (3.16)
	≥34 years	17.8	23.57 (4.31)	6.89 (3.06)	0.96 (3.22)	9.39 (2.77)
Maternal education	<College	69.2	21.98 (3.94)	6.11 (3.22)	0.94 (2.97)	8.58 (3.25)
	≥College	30.8	21.33 (3.49)	6.05 (3.35)	0.94 (2.89)	10.28 (2.86)
Maternal nationality	Taiwan	93.8	22.20 (3.86)	5.99 (3.22) *	0.94 (2.94)	9.21 (3.16)
	others	6.2	16.78 (2.59)	8.67 (4.10)	0.95 (3.03)	7.24 (2.83)
Maternal history of atopy	No	64.9	23.10 (3.97)	6.17 (3.46)	0.86 (2.92)	9.49 (3.13)
	Yes	35.1	20.49 (3.60)	6.11 (3.06)	1.16 (3.00)	8.25 (3.06)
**Children**						
Gender	Male	57.7	23.81 (3.94)	7.69 (3.90)	0.93 (2.97)	9.97 (3.29)
	Female	42.3	21.98 (3.86)	5.93 (3.42)	0.99 (2.89)	8.76 (3.46)
Birth weight	<2500 gm	5.4	15.33 (3.97)	7.46 (2.97)	1.02 (2.72)	7.03 (3.03)
	≥2500 gm	94.6	22.87 (3.74)	6.17 (3.32)	0.92 (2.94)	9.58 (3.22)
Gestational age	<37 weeks	9.0	22.20 (3.29)	6.05 (2.94)	0.70 (2.32)	9.12 (2.64)
	≥37 weeks	91.0	21.98 (3.82)	6.30 (3.35)	0.97 (2.97)	9.30 (3.25)
Parity	<2	82.5	22.65 (3.63)	6.49 (3.35) *	0.96 (2.86) *	9.30 (3.19)
	≥2	17.5	19.30 (4.53)	5.00 (2.97)	0.83 (3.25)	8.25 (2.89)
Breast feeding	No	23.6	17.81 (3.71)	5.93 (3.60)	0.90 (3.25)	8.58 (3.16)
	Yes	76.4	22.65 (3.71)	6.36 (3.25)	0.94 (2.86)	9.39 (3.22)
**Family income per year**						
<600,000 NT dollars		31.3	21.12 (3.53)	6.55 (3.22)	0.91 (2.86)	9.49 (3.19)
600,000–1,500,000 NT dollars		60.7	23.81 (3.94)	6.42 (3.39)	0.96 (3.06)	9.97 (3.29)
>1,500,000 NT dollars		8.0	20.49 (4.31)	6.05 (3.39)	0.99 (3.39)	6.69 (2.72)

GM: geometric mean; MBP: Monobutyl phthalate; MBzP: Monobenzyl phthalate; 5OH-MEHP: Mono(2-ethyl-5-hydroxyhexyl) phthalate; NT dollars: New Taiwan dollars ($1 USD = $33 New Taiwan dollar). * *p* < 0.05.

**Table 2:** Environmental factors of the study population in terms of geometric means (s.e.) of urine phthalate metabolites concentrations in 2010 (*n* = 453).

Environmental Factors		%	MEP (µg/g Creatinine)	MBP (µg/g Creatinine)	MBzP (µg/g Creatinine)	5OH-MEHP (µg/g Creatinine)
Hot food with plastic bags	Seldom	20.5	21.67 (4.07)	6.54 (3.00)	0.96 (3.04)	7.66 (2.73)
	Usually	79.5	25.02 (3.95)	7.15 (3.36)	0.93 (2.82)	11.08 (3.43)
*p* value			0.83	0.40	0.88	0.15
PVC film stored or heated food	Seldom	42.4	19.82 (3.70)	7.24 (3.19)	0.83 (2.73)	9.59 (3.04)
	Usually	57.6	27.82 (4.13)	6.86 (3.35)	1.01 (2.92)	10.46(3.43)
*p* value			0.22	0.73	0.37	0.33
Microwave heated food	Seldom	41.6	21.06 (3.81)	6.08 (2.81)	0.97 (2.67)	9.34 (2.82)
	Usually	58.4	26.58 (4.07)	7.78 (3.58)	0.90 (2.97)	10.85 (3.64)
*p* value			0.29	0.02 *	0.51	0.06
Plastic bottle drinks	Seldom	9.1	13.72 (2.74)	6.10 (2.59)	1.08 (3.58)	9.68 (4.58)
	Usually	90.9	25.53 (4.05)	7.12 (3.34)	0.92 (2.77)	10.25 (3.19)
*p* value			<0.01 *	0.06	0.87	0.51
Plastic nursing bottle, nipple	Seldom	82.4	23.16 (4.54)	7.03 (3.22)	0.93 (2.65)	9.88 (3.32)
	Usually	17.6	25.13 (3.87)	7.18 (3.76)	1.06 (3.85)	12.08 (3.48)
*p* value			0.58	0.76	0.28	0.77
Products containing fragrance	Seldom	97.9	24.15 (3.83)	7.06 (3.29)	0.94 (2.81)	8.72 (2.20)
	Usually	2.1	80.07 (12.25)	7.16 (4.51)	1.44 (5.19)	10.27 (3.38)
*p* value			0.02 *	0.68	0.51	0.65
Wash hands before eating	<2 meals/day	3.7	24.37 (3.90)	7.05 (3.22)	0.93 (2.86)	10.14 (3.24)
	≥2 meals/day	96.3	21.41 (6.28)	6.34 (5.18)	0.69 (1.60)	9.13 (4.38)
*p* value			0.55	0.45	0.04 *	0.76
Instant noodles intake	Seldom	33.4	22.32 (3.99)	6.88 (3.11)	0.88 (2.66)	9.83 (3.25)
	Usually	66.6	25.09 (3.97)	7.32 (3.61)	1.04 (3.19)	10.97 (3.41)
*p* value			0.61	0.18	0.26	0.33
PVC flooring	No	78.6	23.27 (4.14)	6.94 (3.15)	0.88 (2.91)	10.24 (3.30)
	Yes	21.4	24.16 (3.07)	7.53 (3.82)	0.95 (2.86)	10.54 (3.27)
*p* value			0.34	0.21	0.63	0.94
Incense burning at home	<3 times/month	47.3	24.16 (3.95)	6.61 (3.47)	0.91 (2.7)	11.14 (3.49)
	Usually	52.7	22.91 (3.85)	7.50 (3.11)	0.95 (3.01)	9.51 (3.11)
*p* value			0.93	0.65	0.29	0.79
ETS exposure	No	46.8	26.40 (4.14)	6.78 (3.34)	0.89 (2.85)	9.68 (3.36)
	Yes	53.2	22.97 (3.87)	7.49 (3.26)	0.98 (2.91)	10.98 (3.24)
*p* value			0.94	0.92	0.34	0.84
Dehumidifier at home	No	57.9	24.01 (3.77)	7.36 (3.34)	0.94 (2.76)	10.65 (3.33)
	Yes	42.1	23.98 (4.09)	6.82 (3.23)	0.94 (3.05)	9.69 (3.31)
*p* value			0.88	0.42	0.58	0.60

PVC: Polyvinylchloride ; ETS: environmental tobacco smoke. * *p* < 0.05.

**Table 3:** Contaminated food items in terms of geometric means (s.e.) of urine phthalate metabolites concentrations in 2010 (*n* = 453).

DEHP Contaminated Food Intake	%	MEP (µg/g Creatinine)	MBP (µg/g Creatinine)	MBzP (µg/g Creatinine)	5OH-MEHP (µg/g Creatinine)
None	67.9	16.47 (4.24)	4.47 (3.14)	0.59 (2.54)	7.57 (2.66)
Juice, jelly	6.2	16.65 (2.46)	4.63 (2.13)	0.57 (1.86)	7.61 (1.82)
Sport drink	17.7	24.15(4.23)	5.97 (3.36)	0.81 (3.16)	9.23 (1.08)
Probiotics, vitamin supplements	8.1	21.44(2.51)	6.48(2.69)	0.90 (2.86)	19.19 (4.08) *
*p* value		0.77	0.48	0.55	<0.001

* *p* < 0.05.

**Table 4:** Environmental factors of the study population in terms of geometric means (s.e.) of urine phthalate metabolites concentrations in 2013 (*n* = 200).

Environmental Factors		%	MEP (µg/g Creatinine)	MBP (µg/g Creatinine)	MBzP (µg/g Creatinine)	5OH-MEHP (µg/g Creatinine)
Hot food with plastic bags	Seldom	18.0	24.80 (4.35)	6.97 (4.70)	0.91 (2.78)	12.29 (3.64)
	Usually	82.0	25.49 (4.21)	7.02 (4.02)	1.01 (3.26)	12.92 (3.57)
*p* value			0.91	0.56	0.42	0.76
PVC film stored or heated food	Seldom	43.5	24.59 (4.51)	6.61 (3.86)	0.90 (2.83)	11.26 (3.63)
	Usually	56.5	25.98 (4.03)	7.94 (4.48)	0.96 (2.91)	13.36 (3.61)
*p* value			0.82	0.33	0.90	0.32
Microwave heated food	Seldom	46.6	21.49 (3.79)	6.96 (3.84)	0.90 (2.70)	11.16 (3.36)
	Usually	53.4	29.32 (4.57)	7.34 (4.40)	0.95 (3.06)	13.59 (3.85)
*p* value			0.22	0.37	0.37	0.03 *
Plastic bottle drinks	Seldom	10.6	24.82 (4.30)	7.01 (4.17)	0.92 (2.85)	12.36 (3.61)
	Usually	89.4	30.46 (3.64)	8.57 (3.83)	0.99 (2.99)	12.77 (3.84)
*p* value			0.63	0.88	0.96	0.85
Plastic nursing bottle, nipple	Seldom	85.6	22.26 (4.11)	6.62 (4.22)	0.89 (2.75)	11.32 (3.57)
	Usually	14.4	58.97 (4.30)	12.02 (3.62)	1.14 (3.49)	21.01 (4.10)
*p* value			0.12	0.54	0.29	0.18
Products containing fragrance	Seldom	95.4	23.78 (4.04)	6.95 (4.20)	0.71 (1.37)	12.00 (3.60)
	Usually	4.6	120.14 (6.71)	15.48 (3.19)	0.93 (2.91)	32.83 (3.79)
*p* value			<0.01 *	0.72	0.34	0.02 *
Wash hands before eating	<2 meals/day	4.3	41.09 (10.14)	7.20 (4.12)	0.94 (2.90)	32.05 (4.36)
	≥2 meals/day	95.7	24.82 (4.03)	6.34 (4.61)	0.64 (1.83)	12.06 (3.50)
*p* value			<0.01 *	0.80	0.35	0.01 *
Instant noodles intake	Seldom	37.3	25.84 (3.87)	6.21 (4.15)	0.88 (2.83)	10.98 (3.47)
	Usually	62.7	25.09 (4.45)	7.79 (4.10)	1.00 (2.92)	13.33 (3.70)
*p* value			0.47	0.83	0.41	0.26
PVC flooring	No	78.2	24.54 (4.13)	5.12 (3.45)	0.74 (2.45)	10.37 (3.74)
	Yes	21.8	26.84 (3.87)	7.68 (4.31)	0.99 (2.94)	12.91 (3.55)
*p* value			0.84	0.03 *	0.04 *	0.50
Incense burning at home	<3 times/month	51.5	28.78 (3.57)	7.61 (4.43)	0.83 (2.76)	12.03 (3.47)
	Usually	48.5	21.56 (4.57)	6.46 (3.86)	1.04 (2.91)	12.60 (3.74)
*p* value			0.71	0.27	0.52	0.45
ETS exposure	No	52.8	27.40 (4.23)	6.22 (3.88)	1.02 (2.86)	11.51 (3.57)
	Yes	47.2	24.79 (4.23)	8.22 (4.30)	0.81 (2.68)	13.46 (3.70)
*p* value			0.90	0.27	0.15	0.88
Dehumidifier at home	No	55.2	29.53 (4.18)	7.72 (4.29)	0.97 (3.17)	13.34 (3.61)
	Yes	44.8	19.89 (3.89)	6.11 (3.84)	0.90 (2.44)	11.31 (3.46)
*p* value			0.30	0.32	0.09	0.45

PVC: Polyvinylchloride ; ETS: environmental tobacco smoke. * *p* < 0.05.

## Supplementary Materials

The following are available online at www.mdpi.com/1660-4601/14/8/935/s1, Table S1: Selected characteristics of participants in the CEAS cohort.

## Abbreviations

DEP	Diethylphthalate
DBP	Dibutyl phthalate
BBzP	Butylbenzyl phthalate
DEHP	di(2-Ethylhexyl) phthalate
MEP	Monoethyl phthalate
MBP	Monobutyl phthalate
MBzP	Monobenzyl phthalate
5OH-MEHP	Mono(2-ethyl-5-hydroxyhexyl)phthalate
UPLC-MS/MS	Ultra-performance liquid chromatography coupled with tandem mass spectrometry
