# Supplementary material for: Changes in Urinary Phthalate Metabolite Levels Before and After the Phthalate Contamination Event and Identification of Exposure Sources in a Cohort of Taiwanese Children
Source: Int J Environ Res Public Health. 2017 Aug 19;14(8):935. doi: 10.3390/ijerph14080935 (PMC5580637; doi:10.3390/ijerph14080935)
Supplement: Supplementary file 1 [file ijerph-14-00935-s001.docx]

Supplement

**Table S1.**Selected characteristics of participants in the CEAS cohort

|  | With specimens (n=453) | | CEAS cohort(n=3246) | |  |
| --- | --- | --- | --- | --- | --- |
|  | n | % | n | % | p-value |
| ***Mother*** |  |  |  |  |  |
| **Maternal age** ≥34 years (%) | 66 | 14.6 | 503 | 16.6 | 0.545 |
| **Maternal education** ≥College (%) | 118 | 26.0 | 858 | 27.7 | 0.198 |
| **Maternal history of atopy** Yes(%) | 126 | 27.8 | 1205 | 41.4 | 0.022* |
| ***Children*** |  |  |  |  |  |
| **Gender** Male(%) | 261 | 57.6 | 1747 | 54.1 | 0.140 |
| **Birth weight**< 2500 gm(%) | 20 | 4.4 | 196 | 6.6 | 0.385 |
| **Gestational age**<37 weeks (%) | 353 | 77.9 | 2867 | 91.6 | 0.695 |
| **Parity**< 2 (%) | 315 | 69.5 | 2527 | 81.5 | 0.635 |
| ***Environmental factors*** |  |  |  |  |  |
| **Breast feeding**Yes (%) | 292 | 64.5 | 2311 | 74.7 | 0.470 |
| **Incensing at home** Yes (%) | 220 | 48.6 | 1633 | 53.8 | 0.196 |
| **ETS exposure**Yes (%) | 186 | 41.1 | 1781 | 57.8 | 0.650 |
| **Family income per year**†  < 600,000 NT dollars (%) | 110 | 24.3 | 1059 | 32.6 | 0.062 |
| 600,000-1,500,000 NT dollars (%) | 213 | 47.0 | 1542 | 47.5 |  |
| > 1,500,000 NT dollars (%) | 28 | 6.2 | 249 | 7.7 |  |

*New Taiwan dollars per year ($1 USD = $ 33 New Taiwan dollar).

†Number of subjects does not add up to total N because of missing data.

* p < 0.05
